# QuNex Recipes: Executable, Human-Readable Workflows for Reproducible Neuroimaging Research

**DOI:** 10.1101/2025.11.08.687330

**Published:** 2025-11-11

**Authors:** Jure Demšar, Aleksij Kraljič, Andraž Matkovič, Samuel Brege, Lining Pan, Zailyn Tamayo, Clara Fonteneau, Markus Helmer, Jie Lisa Ji, Alan Anticevic, Cole Korponay, Melissa Salavrakos, Matthew F. Glasser, Lisa D. Nickerson, Youngsun T. Cho, Grega Repovš

**Affiliations:** 1 Faculty of Computer and Information Science, University of Ljubljana, Ljubljana, Slovenia; 2 Mind & Brain Lab, Department of Psychology, Faculty of Arts, University of Ljubljana, Ljubljana, Slovenia; 3 Donders Institute for Brain, Cognition, and Behavior, Radboud University, Nijmegen, the Netherlands; 4 Department of Medical Neuroscience, Radboud University Medical Center, Nijmegen, the Netherlands; 5 Department of Psychiatry, Yale University School of Medicine, New Haven, CT, USA; 6 Janssen Research & Development, LLC, a Johnson & Johnson Company, Titusville, NJ, USA; 7 McLean Hospital, Harvard Medical School, Belmont, MA, USA; 8 Department of Radiology, Washington University in St. Louis, St. Louis, MO, USA; 9 Department of Neuroscience, Washington University in St. Louis, St. Louis, MO, USA; 10 Child Study Center, Yale University School of Medicine, New Haven, CT, USA

**Keywords:** neuroimaging, data processing, data analysis, reproducibility

## Abstract

Preprocessing and analysis of neuroimaging data are technically demanding, often requiring a combination of multiple software tools, modality-specific pipelines, and extensive parameter tuning to match dataset characteristics. These complexities make it difficult to document workflows in sufficient detail to ensure complete transparency and reproducibility. To address these challenges, we introduce QuNex recipes, a framework for defining and executing complete neuroimaging workflows – encompassing data onboarding, preprocessing, and analysis – in a transparent, machine- and human-readable format. Recipes are implemented as an integrated feature of the Quantitative Neuroimaging Environment & Toolbox (QuNex), a containerized, open-source platform for end-to-end multimodal and multi-species neuroimaging processing. The recipes framework enables seamless integration of QuNex commands with custom scripts and external tools, capturing every processing step and parameter setting. A fully reproducible study can thus be shared and replicated by providing only (a) the QuNex version used, (b) the recipe file, and (c) the data. This approach standardizes workflow specification, enhances transparency, and enables one-command replication of complex neuroimaging analyses. By providing a standardized way to describe and share workflows, recipes facilitate open exchange of best practices and reproducible methods within the neuroimaging community.

## 1. Introduction

*QuNex (The Quantitative Neuroimaging Environment & Toolbox)* is an open-source software suite that provides an extensible framework for data organization, quality control, preprocessing, and analysis across multiple neuroimaging modalities and species (1). Since its release in 2023, more than 400 institutions and individual users have registered for access. To date, QuNex has been successfully used to process tens of thousands of scans across clinical and large-scale research datasets (2–7), including serving as the primary processing tool for the Human Connectome Project (HCP) team at Washington University for processing most of their recent data releases. QuNex is the only integrated neuroimaging platform that natively incorporates the HCP Minimal Preprocessing Pipelines (8) within a containerised framework that can be easily deployed in a high-performance computing (HPC) environment, enabling reproducible, scalable, and multimodal analyses across human and non-human species.

The original motivation for developing QuNex stemmed from persistent challenges in neuroimaging related to **reproducibility, transparency, and workflow integration** (9–12). In recent years, the neuroimaging community has advanced major initiatives to improve reproducibility, including the **FAIR data principles** (13), **the ReproNim** framework for reproducible neuroimaging (14), and efforts to standardize data and result representations through the **Brain Imaging Data Structure (BIDS)** (15), the **Neuroimaging Data Model (NIDM)** (16), and **DataLad** for distributed data and code management (17). These initiatives have substantially improved how neuroimaging data are organized and shared, yet the reproducibility of *analytic workflows* – the series of steps and parameter choices that transform raw data into scientific results – remains a major bottleneck.

Processing pipelines typically integrate multiple tools (e.g., Connectome Workbench, FreeSurfer, FSL, AFNI, SPM…), each with distinct dependencies and parameter sets. Even when executed within containers that guarantee software version consistency (18, 19), the absence of a unified, human-readable record of the workflow configuration often prevents exact replication. Although several tools address aspects of reproducible neuroimaging research, each has distinct limitations (Table 1). Preprocessing-focused tools like NiPreps (fMRIPrep, sMRIPrep, dMRIPrep) and qsiprep, which are built on the Nipype workflow framework (20), provide robust, containerised pipelines but are limited to preprocessing stages and offer minimal support for external script integration or custom postprocessing analyses (21, 22). Domain-specific platforms such as C-PAC and HALFpipe support end-to-end resting-state workflows but lack flexibility for external tool integration (23, 24). MATLAB-based solutions (DPABI/DPARSF, CONN) provide end-to-end workflows with graphical interfaces, but use binary .mat files that limit version control integration, transparency, and human readability (25, 26). General workflow managers such as Snakemake (27) and Nextflow (28) offer flexible automation and provenance tracking, but require specialized scripting expertise and lack neuroimaging-specific features such as standardised quality control or native support for neuroimaging data organisation standards (BIDS). As a result, reproducing or adapting another group’s analysis often involves extensive manual reconstruction of undocumented steps and parameters, limiting openness and collaboration.

To directly address these challenges, we developed **QuNex Recipes**, a framework that enables fully documented, reproducible definition and execution of neuroimaging workflows. Unlike preprocessing-focused tools, QuNex Recipes supports end-to-end workflows from data onboarding through preprocessing and analysis. Unlike most neuroimaging platforms, it enables seamless external script integration at any workflow stage, not just as pre- or post-processing hooks. It provides native XNAT integration for data management and a four-level hierarchical parameter system for flexible configuration. Importantly, QuNex Recipes achieves this functionality without requiring programming expertise, using human-readable YAML specifications that are version control-friendly and fully transparent. The recipe specification file defines all processing and analysis stages (from data onboarding, preprocessing, and analysis, to quality control) along with the sequence of operations, and exact parameter values. The use of a recipe file within the new QuNex run_recipe command allows every step of a preprocessing and analytic workflow to be encoded in a single, shareable file.

When used together with QuNex’s existing batch processing capabilities, recipes achieve three key goals: (1) **automatic documentation** of all processing and analytic steps within a single file; (2) **flexible aggregation** of diverse preprocessing and analytic workflows within a unified framework; and (3) **shareability and reusability**, allowing collaborators or external researchers to reproduce the full pipeline by providing only (a) the QuNex version used, (b) the recipe file, and (c) the input data.

This design bridges the gap between workflow automation and reproducible reporting, enabling transparent, one-command replication of complex multimodal analyses. By extending the principles of the FAIR and ReproNim movements to executable workflows, QuNex Recipes provide a practical, scalable mechanism for open, collaborative, and fully reproducible neuroimaging research.

## 2. Methods

This section describes the design, and structure of the QuNex recipe framework. We first outline the format used to define recipes and explain how recipes specify each step of a neuroimaging workflow in a transparent and reproducible manner. Next, we detail the process of executing recipes within the containerized QuNex environment, followed by an overview of the internal workflow management system and its integration with high-performance computing resources. Finally, we describe how recipe-based processing can be facilitated for execution within the XNAT (Extensible Neuroimaging Archive Toolkit) environment.

### 2.1 QuNex recipes

QuNex processing recipes are defined using the YAML format (YAML Ain’t Markup Language; https://yaml.org). YAML is a compact and flexible human-readable data serialization standard that can be easily parsed by most programming languages. We selected YAML because it provides an intuitive and transparent structure for defining workflow specifications, while remaining compact and machine-readable. This design enables both non-programmers and advanced users to construct and interpret recipes without additional syntactic overhead.

**Fig. 1.**
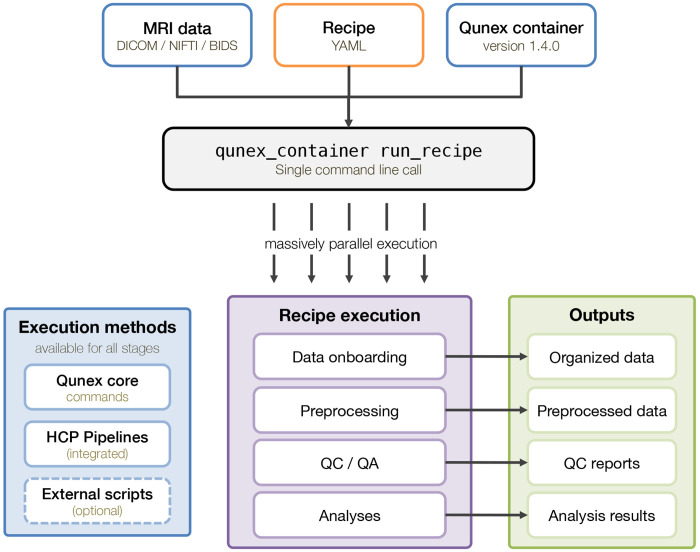
Schematic overview of a QuNex recipe structure. Each recipe file consists of optional global_parameters and one or more defined recipes. Within each recipe, commands are executed sequentially, with parameters inherited hierarchically from global, recipe, or command levels.

Figure 1 illustrates the conceptual flow of a QuNex recipe workflow. A recipe defines all stages of neuroimaging processing – from data onboarding through structural and functional preprocessing to post-processing and analysis – within a single YAML file. Executed inside a versioned QuNex container, the recipe runs all specified commands, quality control steps, and optional external scripts or programs that can be integrated at any stage. Recipes can also be executed in parallel across sessions, enabling scalable processing on local or high-performance compute systems. Together with the data and QuNex version, the recipe forms a complete reproducibility bundle that enables one-command replication of the entire processing and analysis pipeline.

A QuNex recipe contains two main blocks at the root level of the YAML file: global_parameters and recipes. The global_parameters block is optional and defines parameters that apply across all commands listed within the recipes section. The recipes block specifies one or more processing or analysis workflows, each defined as a sequence of commands to be executed.

Each entry within the recipes block represents an individual recipe identified by name. For example, the code snippet in Listing 1 contains two recipes, first_recipe and second_recipe. A recipe may begin with an optional definition of *recipe-level parameters*, which apply only to commands within that recipe. The mandatory commands block defines the ordered sequence of commands to be executed and specifies command-specific parameters. For full flexibility purposes, parameters can be defined at four distinct hierarchical levels:

- command line call parameters,
- command parameters,
- recipe parameters,
- global parameters.

Higher-level parameters take precedence over lower-level ones. When a parameter is defined at multiple levels, the value with the highest precedence is used in the final command call. Parameter values can be provided either statically or dynamically. Dynamic values are injected at runtime through the use of *mustache* notation (double curly braces, {{…}}), which allows substitution from operating system environment variables or external sources. This mechanism supports flexible integration of user-specific or system-dependent values while preserving full reproducibility of workflow specifications.

Listing 1 illustrates the logic and syntax of a typical QuNex recipe, showing the hierarchical organization of parameters, sequential command execution, and invocation of both internal and external processing routines.

**Listing 1.** Example of a QuNex recipe file.

global_parameters:
parameter1: value
parameter2: 100
parameter3: **true**
recipes:
first_recipe:
parameter1: new_value
commands:
– command1:
parameter1: internal_value
– command2
second_recipe:
commands:
– command1:
parameter1: {{ENV_VALUE}}
– external:
path: /scripts/recipe_example.sh
external_parameter: test_value
– command3:
parameter1: some_value

### 2.2 Executing a QuNex recipe

To execute a QuNex recipe (e.g., first_recipe) that is locally stored at /data/qx_recipes/recipe.yaml, the user can invoke the run_recipe command by running the following in a terminal:

qunex_container run_recipe \

 --recipe_file=“/data/qx_recipes/recipe.yaml” \

 --recipe=“first_recipe” \

 --container=“/data/qx_containers/qunex_suite-1.4.0.sif”

This command executes the sequence of QuNex commands defined in the first_recipe using the Singularity/Apptainer container with QuNex version 1.4.0 (the latest version at the time of writing). Additional details about QuNex containerization are available in the official documentation (https://qunex.readthedocs.io).

In this example, command1 is executed with parameter1 equal to the internal_value, as this is the highest-precedence value defined for this parameter. If a parameter is additionally specified on the command line (e.g., -parameter1=“value_x”), that value takes priority, since command-line arguments override all lower-level definitions. Parameters parameter2 and parameter3, defined under global_parameters, are automatically propagated to all commands. Commands that do not utilize a given parameter simply ignore it.

When executing the second_recipe, an environment variable must be defined for ENV_VALUE, which is dynamically injected into parameter1 of command3 through the mustache notation ({{…}}). Environment variables can be set directly through the -bash_post argument, which executes a short shell preamble after (post) entering the container, but before the recipe is launched:

qunex_container run_recipe \

 --recipe_file=“/data/qx_recipes/recipe.yaml” \

 --recipe=“second_recipe” \

 --bash_post=“export_ENV_VALUE=value_injection” \

 --container=“/data/qx_containers/qunex_suite-1.sif”

The second_recipe also illustrates how external scripts, programs, or pipelines can be incorporated into a processing sequence. In this example, QuNex executes the following command as the second processing step:

/scripts/recipe_example.sh --external_parameter=“test_value”

In addition to Bash (.sh) scripts, the recipe framework supports execution of Python and R scripts, as well as precompiled binaries. This flexibility allows integration of custom user code or third-party tools directly within QuNex workflows, while preserving full provenance and reproducibility.

The qunex_container script serves as a unified entry point for executing QuNex commands and recipes. It abstracts interactions with containerization and high-performance computing environments, simplifying workflow execution for users with varying levels of technical expertise and experience with containers and scheduling. Comprehensive documentation of its parameters and capabilities is provided in the original QuNex manuscript (1) and in the online documentation (https://qunex.readthedocs.io).

The examples above demonstrate the general logic and usage of QuNex recipes through simplified placeholders. Practical examples applying recipes to actual MRI datasets are presented in the Results section.

### 2.3 Internal execution and workflow management

When the run_recipe command is invoked, QuNex parses the specified YAML file and constructs an internal representation of the workflow. Each command block is interpreted in sequence, with parameters resolved according to the hierarchical precedence rules described above. This parsing step ensures that all values defined in global_parameters, recipe-level parameters, and command-level parameters are consolidated into a unified parameter space. If dynamic substitution variables (defined via the mustache notation) are detected, they are resolved at runtime by querying the current environment.

After parsing, the recipe is executed through the QuNex workflow engine, which sequentially or concurrently launches processing steps depending on the specified configuration and available compute resources. Each command is executed within the containerized QuNex environment, ensuring consistent access to dependencies and software versions. For computational clusters, the run_recipe framework supports native integration with SLURM, PBS and GridEngine scheduling systems. When executed on a high-performance computing (HPC) system, QuNex automatically generates scheduler submission scripts and manages dependencies between jobs, enabling parallel or batched execution of independent steps.

Each executed command produces structured logs that capture the exact command line invocation, resolved parameter values, execution time, and return status. Logs are stored in a human-readable format to facilitate provenance tracking and post hoc quality control. Upon completion, a summary report is generated, indicating whether all steps completed successfully or if any command terminated with an error. In the latter case, execution can be resumed from the last successful step without re-running completed portions of the workflow. This checkpointing mechanism ensures efficiency and robustness in large-scale processing tasks.

In addition to logging and error handling, the recipe framework records the QuNex container version, execution date, and system environment variables used during runtime. These records provide full provenance metadata, allowing complete replication of the processing environment and facilitating formal reproducibility audits.

Overall, the internal workflow management system enables QuNex recipes to function as self-contained, auditable processing specifications. By combining declarative workflow definitions with containerized execution and automated provenance tracking, QuNex ensures transparency, reproducibility, and scalability across a wide range of computational infrastructures.

### 2.4 XNAT integration

The Extensible Neuroimaging Archive Toolkit (XNAT; Marcus et al. 29) is an open-source imaging informatics platform developed by the Neuroinformatics Research Group at Washington University in St. Louis. XNAT provides a flexible and extensible environment for organizing, storing, and processing neuroimaging data, and supports execution of containerized workflows through its Docker and Singularity/Apptainer integration frameworks. The new recipe-based functionality in QuNex extends this compatibility, enabling streamlined and reproducible data processing directly within XNAT.

There are several advantages to executing QuNex via XNAT. In QuNex, MR sessions are organized in a flat directory structure, allowing efficient concurrent processing with minimal internal parsing. In contrast, XNAT organizes data hierarchically, with sessions nested under subjects and projects. While this structure facilitates data management, it requires that processing be launched on a session-by-session basis. For studies with many sessions, this approach can become inefficient, as the number of individual command calls grows linearly with dataset size. The recipe framework mitigates this limitation by bundling multiple processing and analysis commands into a single executable specification, thereby reducing the number of user interactions required to initiate large-scale processing and minimizing administrative overhead.

A second advantage of running QuNex through XNAT lies in its access control and user management capabilities. QuNex provides a wide range of configurable commands and parameters, which, while powerful, can be complex for new users. Within XNAT, administrators can restrict which commands are available and what data can be accessed, ensuring compliance with project-specific data governance policies. Traditionally, this required administrators to predefine and maintain multiple command configurations – an approach that is both time-consuming and privileges-dependent. By adopting recipe-based processing, administrators now need only authorize a single run_recipe command. Project supervisors and advanced users can then define project-specific recipes containing customized commands, parameters, and checkpoints, all without requiring elevated administrative permissions. This design significantly accelerates user onboarding and reduces the risk of executing incorrect or unauthorized commands.

Finally, XNAT employs a working-directory model, in which archived data are temporarily copied to a processing workspace before execution. Upon successful completion, results are copied back into the archive; failed or incomplete processing runs can be safely discarded, preventing contamination of validated datasets. Although this model enhances data integrity, it can become inefficient for large studies, as each command invocation requires full data transfer. This also complicates iterative reprocessing, since intermediate results may be repeatedly reloaded. The recipe framework addresses this issue by automatically creating *checkpoints* – snapshots of the working directory before and after each processing step. These checkpoints can be recalled during subsequent runs, allowing selective re-execution of failed or updated steps without redundant data transfer. This approach greatly improves both computational efficiency and reproducibility in large-scale or multi-stage analyses.

Overall, integrating QuNex recipes with XNAT provides a unified and efficient mechanism for controlled, reproducible, and scalable processing of neuroimaging data, leveraging the strengths of both platforms in data organization and workflow transparency.

## 3. Results

The Results section illustrates the practical use and reproducibility of the QuNex recipe framework. We first describe the official repository accompanying this manuscript, which contains all materials required to reproduce the examples presented below. In addition, the repository provides an extensive collection of validated recipes that have already been successfully applied in several large-scale and clinical neuroimaging research projects.

We then showcase two representative use cases that demonstrate the flexibility of the recipe framework in real research settings. The first example (Listing 2) showcases an end-to-end workflow beginning with raw MRI data in the DICOM format. The data are converted to NIfTI, onboarded into a QuNex study, processed using the HCP Minimal Preprocessing Pipelines (8), and subsequently analysed for functional connectivity. This use case illustrates how a complete data-processing and analysis pipeline can be encoded and executed through a single recipe, ensuring transparency and full reproducibility.

The second example (Listing 3) highlights the integration of external data sources and specialized diffusion analysis tools within the same framework. Here, preprocessed HCP data (30) are downloaded via an external script, onboarded into a QuNex study, and analysed using FSL’s DTIFit (31) followed by Neurite Orientation Dispersion and Density Imaging (NODDI) microstructure modelling (32, 33). Together, these examples demonstrate how QuNex recipes can support both end-to-end and modular analytic workflows while maintaining complete provenance and reproducibility. Unless otherwise specified, all examples assume that the official QuNex recipe repository is located at /data/qunex_run_recipe and that QuNex studies are stored under /data/studies.

### 3.1 The official QuNex recipe library

The official repository associated with this manuscript (https://github.com/ULJ-Yale/qunex_run_recipe) provides a publicly open and version-controlled library of QuNex recipes, together with all materials required to reproduce the examples presented herein. The repository is organized into the following subfolders:

- example_1 – materials, scripts, and configuration files for the first example described in this manuscript;
- example_2 – materials, scripts, and configuration files for the second example;
- recipes – the main QuNex recipe library containing validated workflows for a variety of processing and analysis scenarios.

The recipe library currently includes a growing collection of tested workflows that have been applied across multiple research projects. These recipes cover a diverse range of use cases, including processing of HCP and HCP-style datasets, legacy data lacking T2-weighted images, imports from DICOM and BIDS formats, longitudinal analyses, diffusion modelling, and functional connectivity workflows.

The repository is designed as a community resource that evolves alongside the QuNex platform. Researchers are invited to extend the library by contributing new or modified recipes through pull requests to the repository. All submissions are reviewed to ensure compatibility and reproducibility before integration into the public collection. This open model supports transparent sharing of analytic best practices and accelerates the dissemination of standardized, reproducible neuroimaging workflows.

### 3.2 A recipe file for end-to-end processing

The first example demonstrates an end-to-end workflow that converts raw MRI data in the DICOM format into preprocessed and analysis-ready outputs suitable for functional connectivity analyses. The recipe includes data onboarding, preprocessing using the HCP Minimal Preprocessing Pipelines (8), HCP ICAFix denoising (34) and HCP MSMAll surface matching (35, 36), running additional functional connectivity preprocessing steps, and computing Global Brain Connectivity (GBC) maps on a combined surface and volume representation across a single resting-state BOLD image. GBC provides a data-driven functional MRI measure that quantifies the average strength of connectivity between each voxel or cortical vertex and all other regions of the brain, providing an index of global network integration (37). This example illustrates how a complete, reproducible processing and analysis workflow can be encapsulated in a single recipe file, enabling transparent documentation and one-command execution.

**Listing 2.** A recipe for end-to-end processing.

global_parameters:
studyfolder: /data/studies/run_recipe_1
sessionsfolder: /data/studies/run_recipe_1/sessions
sessions: pb12280
batchfile: /data/studies/run_recipe_1/processing/batch.txt
recipes:
initial_setup:
commands:
– create_study
import_validate:
commands:
– import_dicom:
add_image_type: 1
– run_qc:
modality: rawNII
outpath: /data/studies/run_recipe_1/sessions/QC/rawNII
customqc: no
omitdefaults: no
scenezip: yes
– run_qa:
datatype: raw_data
configfile: /data/qunex_run_recipe/example_1/qa.yaml
overwrite: yes
hcp_mapping:
commands:
– create_session_info:
mapping: /data/qunex_run_recipe/example_1/hcp_mapping.txt
– create_batch:
overwrite: append
paramfile: /data/qunex_run_recipe/example_1/parameters.txt
hcp_structural:
parameters:
customqc: no
omitdefaults: no
scenezip: yes
commands:
– setup_hcp
– hcp_pre_freesurfer
– hcp_freesurfer
– hcp_post_freesurfer
– run_qc:
modality: T1w
outpath: /data/studies/run_recipe_1/sessions/QC/T1w
– run_qc:
modality: T2w
outpath: /data/studies/run_recipe_1/sessions/QC/T2w
– run_qc:
modality: myelin
outpath: /data/studies/run_recipe_1/sessions/QC/myelin
hcp_functional:
parameters:
bolds: 1
boldsuffix: Atlas
parelements: 1
commands:
– hcp_fmri_volume
– hcp_fmri_surface
– run_qc:
modality: BOLD
outpath: /data/studies/run_recipe_1/sessions/QC/BOLD
scenetemplatefolder: /opt/qunex/qx_library/data/scenes/qc
customqc: no
omitdefaults: no
scenezip: yes
hcp_denoising:
commands:
– hcp_icafix
– hcp_msmall
post_processing:
parameters:
image_target: dtseries
boldsuffix: Atlas
commands:
– map_hcp_data:
hcp_nifti_tail: _MSMAll_hp2000_clean
hcp_cifti_tail: _Atlas_MSMAll_hp2000_clean
– create_bold_brain_masks
– compute_bold_stats
– create_stats_report
– extract_nuisance_signal
– preprocess_bold
compute_gbc:
commands:
– create_list:
bolds: 1
listfile: /data/studies/run_recipe_1/sessions/specs/fc_bolds.list
bold_tail: _Atlas_s_hpss_res-mVWMWB1d_lpss.dtseries.nii
overwrite: yes
check: present
– fc_compute_gbc:
flist: /data/studies/run_recipe_1/sessions/specs/fc_bolds.list
command: mFz:0
targetf: /data/studies/run_recipe_1/analysis/gbc
options: ignore=udvarsme|saveind=all|itargetf=sfolder|verbose=**true**

For illustration purposes, the example specifies a single session (pb12280); however, in typical studies, multiple sessions would be processed, usually by invoking the recipe through a command-line call rather than by manually editing the recipe file. The example recipe is composed of seven distinct sub-recipes, listed in the order in which they should be executed. This structure creates purposeful breakpoints at which users can verify that each stage has completed successfully and that intermediate outputs meet expected quality standards. It also establishes a modular system that facilitates the addition of new recipes for extended processing methods – for example performing seed-based functional connectivity analyses.

The first recipe, initial_setup, is executed once per study or dataset and initializes the folder hierarchy required by QuNex. The remaining recipes are executed for each MRI session and can be grouped into preprocessing and postprocessing stages.

The preprocessing stage includes the import_validate, hcp_mapping, hcp_structural, and hcp_functional recipes. These steps handle the import of raw DICOM files, validation of acquisition parameters and scan mapping, and execution of the HCP Minimal Preprocessing Pipelines. During preprocessing, QuNex automatically generates quality control (QC) snapshots and Connectome Workbench (38) .scene files, enabling both rapid and detailed visual inspections of intermediate results. These QC outputs should be reviewed at each breakpoint before continuing with subsequent stages. Apart from the parameters specified in the QuNex batch file (see (1) and https://qunex.readthedocs.io), these preprocessing steps are largely standardized across studies. In most cases, only minor adjustments – such as modifying the import command for datasets organized in the BIDS format – are required.

The final two recipes, post_processing and calculate_gbc, perform mapping preprocessed data to the QuNex folder structure, denoising and analyses. When mapping the data from HCP to QuNex folder structure for analyses, users can specify which data to use by using the hcp_nifti_tail and hcp_cifti_tail parameters. In our case, we will use minimally preprocessed data, that has been also denoised and sufrace matched on top. Denoising includes regression of nuisance signals derived from ventricular, white matter, and whole-brain masks, together with motion parameters, from the BOLD time series. The resulting residual time series are then used to compute GBC maps. These steps often require project-specific adaptation, which highlights the benefit of the modular recipe design.

This modular approach allows users to customize and extend the workflow while maintaining full transparency and provenance of all analytic steps.

### 3.3 A recipe for starting with existing data

The second example illustrates how QuNex recipes can be used to build upon existing datasets by executing additional diffusion processing steps on already preprocessed data. This use case originates from one of our research projects, in which HCP data were downloaded from a public S3 archive, imported into QuNex, and processed using FSL’s DTIFit (31) and Neurite Orientation Dispersion and Density Imaging (NODDI) microstructure modeling (32, 33).

**Listing 3.** A recipe for DWI NODDI processing of preprocessed HCP data.

global_parameters:
studyfolder: /data/studies/run_recipe_2
sessionsfolder: /data/studies/run_recipe_2/sessions
batchfile: /data/studies/run_recipe_2/processing/batch.txt
sessions: 100307,111716
overwrite: yes
recipes:
dtifit_noddi:
commands:
– create_study
– external:
path: /data/qunex_run_recipe/example_2/aws_hcya.sh
sessions: 100307,111716
source_folder: T1w
target_folder: /data/raw/hcya
access_key: {{$HCP_S3_ACCESS_KEY}}
secret_key: {{$HCP_S3_SECRET_KEY}}
– import_hcp:
inbox: NONE
processed_data: /data/raw/hcya
hcp_dataset: HCYA
– create_batch:
targetfile: /data/studies/run_recipe_2/processing/batch.txt
overwrite: append
– dwi_dtifit
– dwi_noddi_gpu:
cuda_version: 12

This recipe executes six sequential steps across two sessions (HCP Young Adults participants 100307 and 111716). Two sessions are shown here for brevity; however, the same workflow can be readily scaled to thousand sessions or more. By default, QuNex processes sessions sequentially. Parallel processing can be achieved either by launching the recipe simultaneously for multiple sessions or by using a scheduler such as SLURM (https://slurm.schedmd.com/overview.html). When a scheduler is employed, QuNex automatically generates one job per session, enabling efficient distributed execution. For details about the QuNex scheduling framework, please refer to the official QuNex manuscript (1) and documentation https://qunex.readthedocs.io. The example below demonstrates how the recipe can be executed with GPU support through a scheduler:

qunex_container run_recipe \

   --recipe_file=“/data/qunex_run_recipe/example_2/dtifit_noddi.yaml” \

   --recipe=“dtifit_noddi” \

   --nv \

   --scheduler=“SLURM,time=01–00,mem=32G,gres=gpus:1,jobname=qx” \

   --container=“/data/qx_containers/qunex_suite-1.3.1.sif”

The recipe begins with create_study, which initializes a new QuNex study directory and folder structure. The following command, an external script (aws_hcya.sh), downloads the required data from the HCP Amazon S3 bucket (for access instructions, see the HCP Wiki at https://wiki.humanconnectome.org). The next step, import_hcp, onboards the downloaded data into the QuNex study, followed by create_batch, which generates a batch file containing session-specific metadata and processing parameters. For a detailed description of batch files and their purpose, please refer to the official QuNex manuscript (1). These initial steps can be categorized as data onboarding and preparation.

The final two commands, dwi_dtifit and dwi_noddi_gpu, perform the required diffusion processing. The first fits diffusion tensors using FSL’s DTIFit, while the second estimates NODDI parameters using GPU acceleration (indicated by the _gpu suffix). To enable GPU execution, the -nv flag must be specified in the qunex_container call in order to properly load the GPU libraries inside the container, and a GPU must be requested through the scheduler configuration parameter (e.g., gres=gpus:1).

Although only two sessions are shown here, this approach can be scaled to large datasets with minimal modification. In our application, this recipe was used to process the entire HCP Aging dataset – over 1,000 sessions – by executing a single recipe command that automatically handled downloading, onboarding, and diffusion processing. This example demonstrates the scalability of the QuNex recipe framework and its capacity to integrate public datasets, external scripts, and high-performance computing resources into a fully reproducible workflow.

### 3.4 Summary

Together, the two examples demonstrate the flexibility, scalability, and transparency of the QuNex recipe framework. The first example shows that complete end-to-end processing – from raw DICOM data to fully preprocessed and analysed outputs – can be achieved reproducibly through a single, human-readable recipe. The second example illustrates how the same framework can integrate preprocessed data, external scripts, and high-performance computing resources to execute specialized analyses at scale. Across both use cases, all parameters, commands, and software versions are explicitly recorded within the recipe and container environment, ensuring full provenance and facilitating replication of results on any compatible system.

By combining containerized execution, structured parametrization, and a declarative workflow specification, QuNex recipes unify what have traditionally been fragmented stages of neuroimaging analysis. This approach enables researchers to move seamlessly between local, institutional, and cloud computing environments, while maintaining a fully documented and reproducible record of their analytic workflow. In doing so, the recipe framework extends the QuNex platform beyond preprocessing toward a general-purpose system for transparent, scalable, and shareable neuroimaging pipelines.

## 4. Conclusion

QuNex recipes represent a significant advancement toward reproducible, transparent, and scalable processing of neuroimaging data. By providing a standardized, human-readable, and machine-executable format for defining analysis workflows, the framework bridges the long-standing gap between flexibility and reproducibility – two often competing objectives in neuroimaging research.

Through practical examples, we demonstrated how the recipe framework enables complete end-to-end analyses, from on-boarding raw DICOM data to advanced connectivity and diffusion modeling, all within a single YAML-defined workflow. The framework seamlessly integrates containerized QuNex commands with external scripts and analytical tools, ensuring full inter-operability between existing neuroimaging software and custom methods. Once accompanied by its recipe file, QuNex version, and input data, any study can be fully reproduced through a single command-line execution, providing an unprecedented level of reproducibility and transparency.

In addition, QuNex recipes extend the functionality of the XNAT platform by simplifying administrative setup, improving workflow traceability, and reducing opportunities for human error. These features make large-scale neuroimaging pipelines more manageable, lowering the technical barrier for new users while preserving advanced configurability for expert users and system administrators.

Ultimately, the introduction of QuNex recipes has the potential to transform how researchers design, share, and replicate neuroimaging workflows. By encouraging the publication of fully reproducible, executable pipelines, the framework promotes cumulative scientific progress and strengthens confidence in neuroimaging findings. Moving forward, we envision a growing open repository of community-submitted recipes – each representing a transparent, executable record of scientific work – that will serve as a foundation for collaborative, standardized, and reproducible neuroimaging research.

## Figures and Tables

**Table 1.** Comparison of QuNex Recipes with Existing Workflow Management Tools

Feature / Toolbox	QuNex Recipes	NiPreps^a^	qsiprep	C-PAC	HALFpipe	DPABI / DPARSF	CONN Toolbox	Snakemake	Nextflow
Neuroimaging-Specific	Yes	Yes	Yes	Yes	Yes	Yes	Yes	No	No
Config file format	YAML	TOML^b^	TOML^b^	YAML	JSON^c^	MATLAB (.mat)	MATLAB (.mat)	Snakefile, YAML	.config, YAML
Hierarchical Parameters	Yes	Possible^d^	Possible^d^	Yes	No	No	No	Yes	Limited
One-Command Execution	Yes	Yes^e^	Yes	Yes	Limited^f^	Yes	Limited^g^	Yes	Yes
External Scripts Integration	Yes	Partial^h^	No	No	No	No	No	Yes	Yes
Container Integration	Dock/Sing	Dock/Sing	Dock/Sing	Dock/Sing	Dock/Sing	Docker	No	Dock/Sing	Dock/Sing^i^
Multi-Modal Support	F/S/D^j^	F/S/D	D	F	F	F	F	N/A	N/A
Batch Processing	Yes	Yes (sequential)	Yes	Yes	Yes	Yes	Yes	Yes	Yes
HPC/Parallel Execution	Yes	Via Nipype plugin	Via Nipype plugin	Yes	Manual^k^	MATLAB Parallel	Yes	Yes	Yes
XNAT Integration	Yes	Unofficial^l^	No	No	No	No	No	No	No
Checkpoint/Resume	Yes	Yes	Yes	Yes	Yes	No	No	Yes	Yes
Provenance Tracking	QA/QC + logs	HTML + boilerplate	HTML + boilerplate	Logs + HTML QC	Config + logs + HTML	Logs + .mat	.mat	Yes	Yes
End-to-End Workflows	Yes	No^m^	No^m,n^	Partial^o^	Yes	Partial^o^	Yes	Yes	Yes
Quality Control Integration	Yes	Yes	Yes	Yes	Yes	Yes	Yes	Manual	Manual
Programming Required	No	No	No	No	No (GUI)	No (GUI)	No (GUI)	Medium	Medium
Version Control Friendly	Yes	Yes	Yes	Yes	Yes	No	No	Yes	Yes
Reference	(1)	(21)	(22)	(23)	(24)	(25)	(26)	(27)	(28)

*Note.* The table contrasts key features relevant to reproducible neuroimaging research. Docker/Sing. = Docker/Singularity container support.

a NiPreps includes fMRIPrep, sMRIPrep, and dMRIPrep.

b Internally uses TOML, but not exposed to user.

c GUI-generated JSON configuration.

d Possible, but not recommended.

e Separate commands for fMRI and dMRI.

f GUI configuration required first, then CLI execution.

g Every step must be run separately.

h Import of partially preprocessed derivatives.

i Also supports Shifter, Charliecloud, and Sarus.

j F = Functional, S = Structural, D = Diffusion imaging modalities.

k Prepares workload manager scripts.

l Non-official NYUAD plugin available: github.com/XNAT-NYUAD/fmriprep.

m Preprocessing only.

n Reconstruction available via qsirecon.

o Resting-state/connectivity analysis.

## Data Availability

The code and materials needed to reproduce the results and run the recipes can be found in the official open repository of this manuscript: https://github.com/ULJ-Yale/qunex_run_recipe. QuNex is open source. To access the code, please register at https://qunex.yale.edu/registration. You can find a small dataset for testing QuNex functionality in the Quick Start section of the official documentation: https://qunex.readthedocs.io/en/latest/wiki/Overview/QuickStart.html.

## References

[1] Ji Jie Lisa, Demšar Jure, Fonteneau Clara, Tamayo Zailyn, Pan Lining, Kraljič Aleksij, Matkovič Andraž, Purg Nina Helmer Markus, Warrington Shaun, Qunex—an integrative platform for reproducible neuroimaging analytics. Frontiers in Neuroinformatics, 17:1104508, 2023. 37090033 10.3389/fninf.2023.1104508 PMC10113546

[2] Ching Terence HW, Grazioplene Rachael, Bohner Calvin, Kichuk Stephen A, DePalmer Giuliana, D’Amico Elizabeth, Eilbott Jeffrey, Jankovsky Anastasia, Burke Michelle, Hokanson Jamila, Safety, tolerability, and clinical and neural effects of single-dose psilocybin in obsessive–compulsive disorder: protocol for a randomized, double-blind, placebo-controlled, non-crossover trial. Frontiers in Psychiatry, 14:1178529, 2023. 37181888 10.3389/fpsyt.2023.1178529 PMC10166878

[3] Elam Jennifer Stine, Glasser Matthew F, Harms Michael P, Sotiropoulos Stamatios N, Andersson Jesper LR, Burgess Gregory C, Curtiss Sandra W, Oostenveld Robert, Larson-Prior Linda J, Schoffelen Jan-Mathijs, The human connectome project: A retrospective. NeuroImage, page 118543, 2021. 34508893 10.1016/j.neuroimage.2021.118543 PMC9387634

[4] Korponay Cole, Cohen-Gilbert Julia E, Kumar Poornima, Harnett Nathaniel G, Medina Adrian A, Forester Brent P, Ressler Kerry J, Beckmann Christian F, Harper David G, and Nickerson Lisa D. Concurrent large-scale brain dynamics during the emotional face matching task and their relation to behavior and mental health. bioRxiv, pages 2024–09, 2024.

[5] Matkovič Andraž, Anticevic Alan, Murray John D, and Repovš Grega. Static and dynamic fmri-derived functional connectomes represent largely similar information. Network Neuroscience, 7(4):1266–1301, 2023. 38144686 10.1162/netn_a_00325 PMC10631791

[6] Rolls Edmund T, Zhang Ruohan, Deco Gustavo, Vatansever Deniz, and Feng Jianfeng. Selective brain activations and connectivities related to the storage and recall of human object-location, reward-location, and word-pair episodic memories. Human Brain Mapping, 45(15):e70056, 2024. 39436048 10.1002/hbm.70056 PMC11494686

[7] Sun Lianglong, Zhao Tengda, Liang Xinyuan, Xia Mingrui, Li Qiongling, Liao Xuhong, Gong Gaolang, Wang Qian, Pang Chenxuan, Yu Qian, Human lifespan changes in the brain’s functional connectome. Nature neuroscience, pages 1–11, 2025. 10.1038/s41593-025-01907-4 40181189

[8] Glasser Matthew F, Sotiropoulos Stamatios N, Wilson J Anthony, Coalson Timothy S, Fischl Bruce, Andersson Jesper L, Xu Junqian, Jbabdi Saad, Webster Matthew, Polimeni Jonathan R, The minimal preprocessing pipelines for the human connectome project. Neuroimage, 80:105–124, 2013. 23668970 10.1016/j.neuroimage.2013.04.127 PMC3720813

[9] Poldrack Russell A, Baker Chris I, Durnez Joke, Gorgolewski Krzysztof J, Matthews Paul M, Munafò Marcus R, Nichols Thomas E, Poline Jean-Baptiste, Vul Edward, and Yarkoni Tal. Scanning the horizon: towards transparent and reproducible neuroimaging research. Nature reviews neuroscience, 18(2):115–126, 2017. 28053326 10.1038/nrn.2016.167 PMC6910649

[10] Laird Angela R. Large, open datasets for human connectomics research: Considerations for reproducible and responsible data use. NeuroImage, 244:118579, 2021. 34536537 10.1016/j.neuroimage.2021.118579

[11] Marek Scott, Tervo-Clemmens Brenden, Calabro Finnegan J, Montez David F, Kay Benjamin P, Hatoum Alexander S, Donohue Meghan Rose, Foran William, Miller Ryland L, Hendrickson Timothy J, Reproducible brain-wide association studies require thousands of individuals. Nature, pages 1–7, 2022. 10.1038/s41586-022-04492-9 PMC8991999 35296861

[12] Botvinik-Nezer Rotem, Holzmeister Felix, Camerer Colin F, Dreber Anna, Huber Juergen, Johannesson Magnus, Kirchler Michael, Iwanir Roni, Mumford Jeanette A, Adcock R Alison, Variability in the analysis of a single neuroimaging dataset by many teams. Nature, 582(7810):84–88, 2020. 32483374 10.1038/s41586-020-2314-9 PMC7771346

[13] Wilkinson Mark D, Dumontier Michel, Aalbersberg IJsbrand Jan, Appleton Gabrielle, Axton Myles, Baak Arie, Blomberg Niklas, Boiten Jan-Willem, Santos Luiz Bonino da Silva, Bourne Philip E, The fair guiding principles for scientific data management and stewardship. Scientific data, 3(1):1–9, 2016. 10.1038/sdata.2016.18 PMC4792175 26978244

[14] Kennedy David N, Abraham Sanu A, Bates Julianna F, Crowley Albert, Ghosh Satrajit, Gillespie Tom, Goncalves Mathias, Grethe Jeffrey S, Halchenko Yaroslav O, Hanke Michael, Everything matters: the repronim perspective on reproducible neuroimaging. Frontiers in neuroinformatics, 13:1, 2019. 30792636 10.3389/fninf.2019.00001 PMC6374302

[15] Gorgolewski Krzysztof J, Auer Tibor, Calhoun Vince D, Craddock R Cameron, Das Samir, Duff Eugene P, Flandin Guillaume, Ghosh Satrajit S, Glatard Tristan, Halchenko Yaroslav O, The brain imaging data structure, a format for organizing and describing outputs of neuroimaging experiments. Scientific data, 3(1):1–9, 2016. 10.1038/sdata.2016.44 PMC4978148 27326542

[16] Maumet Camille, Auer Tibor, Bowring Alexander, Chen Gang, Das Samir, Flandin Guillaume, Ghosh Satrajit, Glatard Tristan, Gorgolewski Krzysztof J, Helmer Karl G, Sharing brain mapping statistical results with the neuroimaging data model. Scientific data, 3(1):1–15, 2016. 10.1038/sdata.2016.102 PMC5139675 27922621

[17] Halchenko Yaroslav O, Meyer Kyle, Poldrack Benjamin, Solanky Debanjum Singh, Wagner Adina S, Gors Jason, MacFarlane Dave, Pustina Dorian, Sochat Vanessa, Ghosh Satrajit S, Datalad: distributed system for joint management of code, data, and their relationship. Journal of Open Source Software, 6(63):3262, 2021. 39469147 10.21105/joss.03262 PMC11514317

[18] Boettiger Carl. An introduction to docker for reproducible research. ACM SIGOPS Operating Systems Review, 49(1):71–79, 2015.

[19] Gregory M Kurtzer, Sochat Vanessa, and Bauer Michael W. Singularity: Scientific containers for mobility of compute. PloS one, 12(5):e0177459, 2017. 28494014 10.1371/journal.pone.0177459 PMC5426675

[20] Gorgolewski Krzysztof, Burns Christopher D, Madison Cindee, Clark Dav, Halchenko Yaroslav O, Waskom Michael L, and Ghosh Satrajit S. Nipype: a flexible, lightweight and extensible neuroimaging data processing framework in python. Frontiers in neuroinformatics, 5:13, 2011. 21897815 10.3389/fninf.2011.00013 PMC3159964

[21] Esteban Oscar, Markiewicz Christopher J, Blair Ross W, Moodie Craig A, Isik A Ilkay, Erramuzpe Asier, Kent James D, Goncalves Mathias, DuPre Elizabeth, Snyder Madeleine, fmriprep: a robust preprocessing pipeline for functional mri. Nature methods, 16(1):111–116, 2019. 30532080 10.1038/s41592-018-0235-4 PMC6319393

[22] Cieslak Matthew, Cook Philip A., He Xiaosong, Yeh Fang-Cheng, Dhollander Thijs, Adebimpe Azeez, Aguirre Geoffrey K., Bassett Danielle S., Betzel Richard F., Bourque Josiane, Cabral Laura M., Davatzikos Christos, Detre John A., Earl Eric, Elliott Mark A., Fadnavis Shreyas, Fair Damien A., Foran Will, Fotiadis Panagiotis, Garyfallidis Eleftherios, Giesbrecht Barry, Gur Ruben C., Gur Raquel E., Kelz Max B., Keshavan Anisha, Larsen Bart S., Luna Beatriz, Mackey Allyson P., Milham Michael P., Oathes Desmond J., Perrone Anders, Pines Adam R., Roalf David R., Adam Richie-Halford Ariel Rokem, Sydnor Valerie J., Tapera Tinashe M., Tooley Ursula A., Vettel Jean M., Yeatman Jason D., Grafton Scott T., and Satterthwaite Theodore D. QSIPrep: An integrative platform for preprocessing and reconstructing diffusion MRI data. Nature Methods, 18(7):775–778, July 2021. doi: 10.1038/s41592-021-01185-5. 34155395 PMC8596781

[23] Cameron Craddock, Sharad Sikka, Brian Cheung, Ranjeet Khanuja, Satrajit Ghosh, Chaogan Yan, Qingyang Li, Daniel Lurie, Joshua Vogelstein, Randal Burns, Stanley Colcombe, Maarten Mennes, Clare Kelly, Di Martino Adriana Castellanos Francisco, and Michael Milham. Towards Automated Analysis of Connectomes: The Configurable Pipeline for the Analysis of Connectomes (C-PAC). Frontiers in Neuroinformatics, 7, 2013. doi: 10.3389/conf.fninf.2013.09.00042.

[24] Waller Lea, Erk Susanne, Pozzi Elena, Toenders Yara J., Haswell Courtney C., Marc Büttner, Thompson Paul M, Schmaal Lianne, Morey Rajendra A., Walter Henrik, and Veer Ilya M. ENIGMA HALFpipe : Interactive, reproducible, and efficient analysis for resting-state and task-based fMRI data. Human Brain Mapping, 43(9):2727–2742, June 2022. doi: 10.1002/hbm.25829. 35305030 PMC9120555

[25] Yan Chao-Gan, Wang Xin-Di, Zuo Xi-Nian, and Zang Yu-Feng. DPABI: Data Processing & Analysis for (Resting-State) Brain Imaging. Neuroinformatics, 14(3):339–351, July 2016. doi: 10.1007/s12021-016-9299-4. 27075850

[26] Whitfield-Gabrieli Susan and Nieto-Castanon Alfonso. *Conn* : A Functional Connectivity Toolbox for Correlated and Anticorrelated Brain Networks. Brain Connectivity, 2(3):125–141, June 2012. doi: 10.1089/brain.2012.0073. 22642651

[27] Köster Johannes and Rahmann Sven. Snakemake—a scalable bioinformatics workflow engine. Bioinformatics, 28(19):2520–2522, 2012. 22908215 10.1093/bioinformatics/bts480

[28] Tommaso Paolo Di, Chatzou Maria, Floden Evan W, Barja Pablo Prieto, Palumbo Emilio, and Notredame Cedric. Nextflow enables reproducible computational workflows. Nature biotechnology, 35 (4):316–319, 2017. 10.1038/nbt.3820 28398311

[29] Marcus Daniel S., Olsen Timothy R., Ramaratnam Mohana, and Buckner Randy L. The extensible neuroimaging archive toolkit. Neuroinformatics, 5:11–34, 2007. 17426351 10.1385/ni:5:1:11

[30] Van Essen David C, Smith Stephen M, Barch Deanna M, Behrens Timothy EJ, Yacoub Essa, Ugurbil Kamil, Wu-Minn HCP Consortium, The wu-minn human connectome project: an overview. Neuroimage, 80:62–79, 2013. 23684880 10.1016/j.neuroimage.2013.05.041 PMC3724347

[31] Jenkinson Mark, Beckmann Christian F, Behrens Timothy EJ, Woolrich Mark W, and Smith Stephen M. Fsl. Neuroimage, 62(2):782–790, 2012. 21979382 10.1016/j.neuroimage.2011.09.015

[32] Zhang Hui, Schneider Torben, Wheeler-Kingshott Claudia A, and Alexander Daniel C. Noddi: practical in vivo neurite orientation dispersion and density imaging of the human brain. Neuroimage, 61(4):1000–1016, 2012. 22484410 10.1016/j.neuroimage.2012.03.072

[33] Hernandez-Fernandez Moises, Reguly Istvan, Jbabdi Saad, Giles Mike, Smith Stephen, and Sotiropoulos Stamatios N. Using gpus to accelerate computational diffusion mri: From microstructure estimation to tractography and connectomes. Neuroimage, 188:598–615, 2019. 30537563 10.1016/j.neuroimage.2018.12.015 PMC6614035

[34] Salimi-Khorshidi Gholamreza, Douaud Gwenaëlle, Beckmann Christian F, Glasser Matthew F, Griffanti Ludovica, and Smith Stephen M. Automatic denoising of functional mri data: combining independent component analysis and hierarchical fusion of classifiers. Neuroimage, 90:449–468, 2014. 24389422 10.1016/j.neuroimage.2013.11.046 PMC4019210

[35] Robinson Emma C, Garcia Kara, Glasser Matthew F, Chen Zhengdao, Coalson Timothy S, Makropoulos Antonios, Bozek Jelena, Wright Robert, Schuh Andreas, Webster Matthew, Multimodal surface matching with higher-order smoothness constraints. Neuroimage, 167:453–465, 2018. 29100940 10.1016/j.neuroimage.2017.10.037 PMC5991912

[36] Robinson Emma C, Jbabdi Saad, Glasser Matthew F, Andersson Jesper, Burgess Gregory C, Harms Michael P, Smith Stephen M, Van Essen David C, and Jenkinson Mark. Msm: a new flexible framework for multimodal surface matching. Neuroimage, 100:414–426, 2014. 24939340 10.1016/j.neuroimage.2014.05.069 PMC4190319

[37] Cole Michael W, Yarkoni Tal, Repovš Grega, Anticevic Alan, and Braver Todd S. Global connectivity of prefrontal cortex predicts cognitive control and intelligence. Journal of Neuroscience, 32(26):8988–8999, 2012. 22745498 10.1523/JNEUROSCI.0536-12.2012 PMC3392686

[38] Marcus Daniel S, Harms Michael P, Snyder Abraham Z, Jenkinson Mark, Wilson J Anthony, Glasser Matthew F, Barch Deanna M, Archie Kevin A, Burgess Gregory C, Ramaratnam Mohana, Human connectome project informatics: quality control, database services, and data visualization. Neuroimage, 80:202–219, 2013. 23707591 10.1016/j.neuroimage.2013.05.077 PMC3845379

